# Bile-Salt-Hydrolases from the Probiotic Strain *Lactobacillus johnsonii* La1 Mediate Anti-giardial Activity *in Vitro* and *in Vivo*

**DOI:** 10.3389/fmicb.2017.02707

**Published:** 2018-01-31

**Authors:** Thibault Allain, Soraya Chaouch, Myriam Thomas, Isabelle Vallée, André G. Buret, Philippe Langella, Philippe Grellier, Bruno Polack, Luis G. Bermúdez-Humarán, Isabelle Florent

**Affiliations:** ^1^Commensal and Probiotics-Host Interactions Laboratory, Micalis Institute, Institut National de la Recherche Agronomique, AgroParisTech, Jouy-en-Josas, France; ^2^UMR7245, Muséum National d'Histoire Naturelle, Centre National de la Recherche Scientifique, Sorbonne-Universités, Paris, France; ^3^JRU BIPAR, ANSES, Ecole Nationale Vétérinaire d'Alfort, INRA, Université Paris-Est, Animal Health Laboratory, Maisons-Alfort, France; ^4^Department of Biological Sciences, University of Calgary, Calgary, AB, Canada; ^5^JRU BIPAR, Ecole Nationale Vétérinaire d'Alfort, ANSES, INRA, Université Paris-Est, Maisons-Alfort, France

**Keywords:** *Giardia duodenalis*, lactobacilli, *Lactobacillus johnsonii*, bile salt hydrolases, BSH, conjugated bile salts, anti-giardial activity

## Abstract

*Giardia duodenalis* (syn. *G. lamblia, G. intestinalis*) is the protozoan parasite responsible for giardiasis, the most common and widely spread intestinal parasitic disease worldwide, affecting both humans and animals. After cysts ingestion (through either contaminated food or water), *Giardia* excysts in the upper intestinal tract to release replicating trophozoites that are responsible for the production of symptoms. In the gut, *Giardia* cohabits with the host's microbiota, and several studies have revealed the importance of this gut ecosystem and/or some probiotic bacteria in providing protection against *G. duodenalis* infection through mechanisms that remain incompletely understood. Recent findings suggest that Bile-Salt-Hydrolase (BSH)-like activities from the probiotic strain of *Lactobacillus johnsonii* La1 may contribute to the anti-giardial activity displayed by this strain. Here, we cloned and expressed each of the three *bsh* genes present in the *L. johnsonii* La1 genome to study their enzymatic and biological properties. While BSH47 and BSH56 were expressed as recombinant active enzymes, no significant enzymatic activity was detected with BSH12. *In vitro* assays allowed determining the substrate specificities of both BSH47 and BSH56, which were different. Modeling of these BSHs indicated a strong conservation of their 3-D structures despite low conservation of their primary structures. Both recombinant enzymes were able to mediate anti-giardial biological activity against *Giardia* trophozoites *in vitro*. Moreover, BSH47 exerted significant anti-giardial effects when tested in a murine model of giardiasis. These results shed new light on the mechanism, whereby active BSH derived from the probiotic strain *Lactobacillus johnsonii* La1 may yield anti-giardial effects *in vitro* and *in vivo*. These findings pave the way toward novel approaches for the treatment of this widely spread but neglected infectious disease, both in human and in veterinary medicine.

## Introduction

*Giardia duodenalis* (syn. *Giardia lamblia* and *Giardia intestinalis*) is a flagellated protozoan parasite responsible for giardiasis, an intestinal zoonotic disease infection that may cause acute or chronic diarrhea, weight loss, malabsorption, abdominal pain, and nausea (Ankarklev et al., 2010; Cotton et al., 2011). It is one of the most common intestinal parasites and one of the most frequent causes of diarrhea, with over 280 million human symptomatic cases worldwide (Lane and Lloyd, 2002; Platts-Mills et al., 2015). Infections occur mainly by the ingestion of cysts present in contaminated food and water. After ingestion, infectious cysts differentiate into trophozoite stages, which in turn colonize the upper small intestine. Included in the “Neglected Disease Initiative” of the World Health Organization (WHO) in 2004, giardiasis has a significant public health impact in both developed and developing countries (Savioli et al., 2006; Platts-Mills et al., 2015). Metronidazole is the most frequently used drug for treating *G. duodenalis* infections, whereas albendazole, tinidazole, and nitazoxanide may also be used with efficacy (Gardner and Hill, 2001; Petri, 2005). Although these drugs have different modes of action, there is an increasing incidence of parasite resistance, and treatment failure is relatively common (Ansell et al., 2015). Moreover, these standard treatments are commonly associated with undesirable side effects in both medical and veterinary usages (Barr et al., 1994; Gardner and Hill, 2001). A successful vaccine has proven elusive, and *Giardia* is able to escape host immunity by switching its variant-specific surface proteins (Singer et al., 2001). Together, these observations underscore the need for new therapeutic alternatives for the treatment of giardiasis.

In the last decade, some probiotics (i.e., live microorganisms which, when administered in adequate amounts, confer a health benefit on their hosts, WHO 2001), in particular several species belonging to the genus *Lactobacillus*, have shown anti-giardial efficacy in various murine models (see Travers et al., 2011 for review). The mechanisms remain incompletely understood but may involve host immunomodulation and/or extracellular compounds released by the bacteria (Perez et al., 2001; Humen et al., 2005; Shukla et al., 2008; Shukla and Sidhu, 2011; Goyal et al., 2013). In this context, we have recently shown that unconjugated bile salts, generated by secreted or released enzymes by the probiotic strain of *Lactobacillus johnsonii* La1 (also known as *L. johnsonii* NCC533), may contribute to the inhibition of *Giardia* trophozoite growth *in vitro* (Perez et al., 2001; Travers et al., 2016). BSH (also called cholylglycine hydrolase, EC 3.5.1.2) are enzymes that hydrolyze the amide bond of conjugated bile salts, liberating the amino acid moiety from the steroid core and generating deconjugated bile salts (i.e., cholic acid, deoxycholic acid and chenodeoxycholic acid) (Begley et al., 2006). Conjugated-bile salts are synthesized in the liver where conjugation to either glycine or taurine occurs, and these conjugated-bile salts play an important role in the solubility and absorption of lipids and cholesterol in the intestinal tract (Eyssen, 1973; Kim et al., 2005; Begley et al., 2006). Moreover, glyco- and tauro-conjugated bile salts exert detergent and antimicrobial properties (Ruiz et al., 2013). BSH activities lead to bile salt detoxification and confer a competitive advantage to the microbial communities that express them, such as lactobacilli in the upper part of the small intestine (Ridlon et al., 2006; Ruiz et al., 2013).

In this study, we cloned and expressed each one of the three-*bsh* genes (i.e., *bsh12, bsh47*, and *bsh56*) from *L. johnsonii* La1 (Pridmore et al., 2004) in *Escherichia coli* in order to evaluate their substrate specificities and to assess their anti-*Giardia* activities, both *in vitro* and *in vivo*. A comparative structural analysis of the three BSHs was also performed using *in silico* approaches to explore whether structural differences could explain possible differences in substrate specificities. The three recombinant BSHs, rBSH12, rBSH47, and rBSH56, were tested against two different strains of the human assemblage A of *G. duodenalis* (WB6 and NF) *in vitro*. Then, rBSH47 was selected to be tested *in vivo* on OF1 suckling mice infected with the *G. duodenalis* strain WB6.

## Materials and methods

### *In silico* analysis of BSHs

Bile salt hydrolases amino acid sequences from different bacterial species were retrieved from databases using BLASTP program from the National Center for Biotechnology Information (NCBI, http://www.ncbi.nlm.nih.gov/) and analyzed *in silico*. Multiple sequence alignments of BSH amino acid sequences were performed using CLUSTALO 1.2.1 (http://www.ebi.ac.uk/Tools/msa/clustalo/) to identify the conserved motifs between the different enzymes. Phylogenetic relationships and phylogenic clustering of BSHs from different species were established by neighbor-joining methods using MEGA5 software (http://www.megasoftware.net/; Tamura et al., 2011). Three-dimensional modeling of *L. johnsonii* La1-BSHs was performed using I-TASSER from University of Michigan (http://zhanglab.ccmb.med.umich.edu/I-TASSER/; Roy et al., 2010). According to C-score results, the BSH from *Bifidobacterium longum* (Kumar et al., 2006) was chosen as template for modeling BSH56, whereas the BSH from *Clostridium perfringens* was used as template for modeling both BSH12 and BSH47 (Rossocha et al., 2005). Models for structure predictions were selected according to the highest values of their C-score (measured for evaluating global and local similarity between query and template protein). Protein structure analysis was performed using Pymol (PyMOL Molecular Graphics System, Version 1.8 Schrödinger, LLC).

### Bacteria and growth conditions

*Lactobacillus johnsonii* La1 strain (Pridmore et al., 2004) was cultured in Man Rogosa Sharpe broth (MRS, Difco) and grown at 37°C in an anaerobic jar using BBL GasPak Anaerobic System (BD), incubated overnight (ON). *E. coli* TOP10 chemically competent cells (Invitrogen) were used for the subcloning of PCR fragments. *E. coli* CYS21 and SE1 chemically competent strains (DelphiGenetic, Belgium) were used, respectively, for the cloning and expression of BSHs. *E. coli* strains were grown in Luria-Bertani (LB) medium at 37°C ON with vigorous shaking at 180 rpm. All bacterial strains were stored at −80°C with 15% (v/v) glycerol for cryoprotection.

### Cloning of *bsh* genes from *L. johnsonii* La1

Genomic DNA of *L. johnsonii* La1 was extracted from 2 mL of an ON culture using Wizard Genomic DNA Purification Kit Protocol (Promega) and used as template to amplify the 3 *bsh* genes: *bsh12* (Gene ID: 2743525), *bsh47* (Gene ID: 2743183), and *bsh56* (Gene ID: 2743142) (Pridmore et al., 2004). The coding sequences of *bsh*12, *bsh*47, and *bsh*56 genes (excluding the putative signal sequences) were amplified by PCR (Phusion Taq, Thermo Fisher Scientific) using primers described in Table 1. These primers were designed to incorporate two restriction sites: *Nhe*I (forward primer) and *Xho*I (reverse primer). The amplified PCR fragments were purified using SV Gel and PCR Clean-Up System (Wizard) and were subcloned into the vector pCR®2.1-TOPO® (Invitrogen). The resulting constructions (pLB487, pLB488, and pLB489) were validated by sequencing (MWG-Genomic Company, Germany) before recovering the *bsh* genes with *Nhe*I and *Xho*I restriction enzymes and cloning them into pStaby 1.2 vector (DelphiGenetics) previously digested with the same enzymes. The pStaby 1.2 plasmid was used for intermediate cloning to introduce a C-terminal six-Histidine tag (His-tag), allowing subsequent purification of rBSHs using affinity chromatography. The resulting plasmids were transferred into *E. coli* CYS21 strains and transformants were grown at 37°C ON in 10 mL of LB containing ampicillin (Amp, 100 μg/ml) with shaking at 180 rpm. Plasmid DNA were extracted from positive clones, sequenced to confirm identity, and subsequently transformed into *E. coli* SE1 expression strain. Bacterial strains, plasmids, and primer sequences used in this study are described in Table 1. Immunoblotting experiments were performed on *E. coli* SE1 (pLB490), *E. coli* SE1 (pLB491), and *E. coli* SE1 (pLB492) strains lysates (see below) using mouse monoclonal 6x-His Epitope Tag Antibody (Thermo Fisher Scientific) to detect recombinant BSHs.

**Table 1:** Bacterial strains, plasmids, primers used in this study.

**Material**	**Relevant properties**	**Source/reference**
**STRAINS**		
***L. johnsonii***		
La1 (NCC533)	Wild type strain	Nestlé collection center NCC533
***E. coli***		
TOP10	Cloning strain	Invitrogen
CYS21	Cloning strain	DelphiGenetics
SE1	Expression strain	DelphiGenetics
**PLASMIDS**		
pCR2.1-TOPO	^*^Amp^r^, subcloning vector	Invitrogen
pLB487	^*^Amp^r^, TOPO harboring *bsh12* gene	This study
pLB488	^*^Amp^r^, TOPO harboring *bsh47* gene	This study
pLB489	^*^Amp^r^, TOPO harboring *bsh56* gene	This study
pStaby express	Amp^r^, subcloning vector	DelphiGenetics
pLB490	^*^Amp^r^, pStaby harboring *bsh12* gene	This study
pLB491	^*^Amp^r^, pStaby harboring *bsh47* gene	This study
pLB492	^*^Amp^r^, pStaby harboring *bsh56* gene	This study
**Primers**	Sequence	
bsh12Fw	5′CCGCTAGCTGTACCTCAATTGTTTATAGTTC 3′	This study
bsh12Rev	3′GGCTCGAGATTTTGATAATTAATTGTTTGC 5′	This study
bsh47Fw	5′CCGCTAGCTGTACTGGTTTAAGATTCAC 3′	This study
bsh47Rev	3′GGCTCGAGGTAAGTCACAAGACTGGTTG 5′	This study
bsh56Fw	5′CCGCTAGCTGTACATCAATTTTATATAGTCC 3′	This study
bsh56Rev	3′GGCTCGAGATTTTCAAATTTAATGGCTTG 5′	This study

### Expression and purification of recombinant BSH12, BSH47, and BSH56 in *E. coli*

*E. coli* SE1 strains harboring pLB490 (*bsh12*), pLB491 (*bsh47*), and pLB492 (*bsh56*) were grown at 37°C ON in 10 mL of LB supplemented with ampicillin (100 μg/mL) with vigorous shaking at 180 rpm and subsequently grown in 1.5 L of LB/ampicillin (100 μg/mL) at 37°C. When an optical density (OD_600nm_) = 0.6–0.8 was reached, gene expression was induced by the addition of 1 mM of Isopropyl β-D-1-Thiogalactopyranoside (IPTG), and cultures were incubated at 21°C ON with shaking at 180 rpm. Bacteria were harvested by centrifugation and cell pellets were washed with PBS and resuspended in 15 ml of Tris-KCl buffer (Tris 50 mM, KCl 100 mM, MgCl_2_ 10 mM, pH 7.5) supplemented with Triton-X-100 (Sigma-Aldrich) to a final concentration of 1% and protease inhibitors 1X (Roche). Cells were subsequently sonicated for 4 min with alternated pulses on ice (on: 5 s, off: 30 s). The lysed cells were then placed in ultracentrifuge tubes and spun at 220,000 × g at 4°C for 45 min to separate soluble supernatants from pellets.

The soluble fractions containing the recombinant BSH (rBSH) were then collected and rBSHs were purified using affinity chromatography. Briefly, columns kept in nickel-nitrilotriacetic acid (Ni-NTA; Qiagen) agarose were first washed with milliQ water and equilibrated with 50 mM Tris-KCl buffer pH 7.5 according to the supplier's protocol. Soluble lysates were passed through Ni-NTA columns and washed with 50 mM Tris-KCl buffer pH 7.5 to remove unbound proteins. Finally, rBSHs were eluted by increasing imidazole concentrations (25, 75, and 500 mM) as recommended by the supplier. The eluted proteins were desalted using Sephadex G-25 columns (Amersham Biosciences). All fractions were analyzed on Sodium Dodecyl Sulfate-PolyAcrylamide Gel Electrophoresis (SDS-PAGE) and stained with Coomassie Brilliant Blue.

### Bile salt hydrolase activity assays

The substrate specificity of each rBSHs was assessed on plates using an agar test. *E. coli* strains harboring pLB490, pLB491, and pLB492 were cultured in LB broth in presence of ampicillin (100 μg/ml). Overnight cultures were then spotted on LB agar plates supplemented with either 0.5% taurodeoxycholic acid (TDCA, Sigma-Aldrich) or 0.5% glycodeoxycholic acid (GDCA, Merck Millipore) and incubated at 37°C for 48 h.

The BSH hydrolyzing activities were also monitored using purified recombinant enzymes in presence of conjugated bile salts, in solution, by measuring the liberation of amino acids (glycine or taurine) as previously described (Grill et al., 2000). A volume of 100 μl of rBSH (20 μg) was mixed with 100 μl of 2.4 g/L of each conjugated bile salts (GDCA, TDCA, glycocholic acid, or taurocholic acid) and incubated for 30 min at 37°C. BSH from *C. perfringens* (Sigma-Aldrich, reference C4018) was used as a positive control. A solution without bile salts was used as a negative control. The hydrolysis of bile salts was stopped by adding 200 μl of 15% trichloroacetic acid (TCA) (v/v%) and the mixture was spun at 10,000 g for 15 min to remove precipitated proteins. The supernatant (80 μl) was subsequently collected and added to 680 μl of 0.3 M borate buffer, 1% SDS (pH 9.5), and 80 μl of 0.3% picrylsulfonic acid solution (Sigma-Aldrich). Mixtures were incubated for 30 min in the dark at room temperature and 800 μL of 0.6 mM HCl was added to stop the colorimetric reaction. The amount of glycine or taurine released was measured at 416 nm using a spectrophotometer and standard curves were established with free glycine and taurine.

### *Giardia duodenalis* cultures

Two different isolates of assemblage A were used in this study: *G. duodenalis* strains WB clone 6 (WB6, ATCC50803), isolated from a patient with chronic giardiasis, and *G. duodenalis* NF (kindly provided by Dr. André Buret, University of Calgary), obtained from an outbreak of human giardiasis. Trophozoites were cultured in axenic conditions grown in Keiser's modified TYI-S-33 medium (KM) adjusted at pH 6.0 and supplemented with heat-inactivated fetal calf serum (10%) (FCS, reference A15-101, PAA laboratories, GE Healthcare) as recently described (Travers et al., 2016). *In vitro* experiments were performed with or without bovine bile (Difco, DB Diagnostic System, reference 212820) supplementation (0.6 g/L).

### Anti-giardial activity assays

Increasing concentrations of rBSHs were co-incubated with fresh cultures of *G. duodenalis* WB6 trophozoites (2 × 10^5^ parasites/ml) in KM medium supplemented with 10% FCS in a final volume of 480 μl, at 37°C in anaerobic conditions for 22 h. Experiments were performed with or without bovine bile (0.6 g/L) supplementation. BSH from *C. perfringens* (1U, Sigma-Aldrich, reference C4018) was used as a positive control. Trophozoites were detached from tubes by chilling on ice for 10 min and the parasite load was measured by using hemocytometer (flagella mobility was used as viability criteria). The inhibition levels were determined in comparison with values of non-treated trophozoite cultures (percentage of growth). Three biological replicates were performed, each in duplicates. The half maximal inhibitory concentrations (IC_50_) were calculated using Prism 5 software (GraphPad).

### *G. duodenalis* viability assays on cell cultures

Caco-2 epithelial cells (human colonic adenocarcinoma, ATTC HTB-37) were grown in Dulbecco's Modified Eagle's Medium (DMEM) containing 200 mM L-glutamine, 100 U/mL penicillin, 100 U/mL streptomycin, and 10% fetal bovine serum (FBS) (Gibco, reference 12484-028) at 37°C and 5% CO_2_. Caco-2 cells were cultured (passage 28–32) at 80% confluence with trypsin-EDTA and seeded at 10^5^ cells/mL onto 12-wells plates (Caco-2 growth medium). Cells were cultured until the monolayer was confluent (3–4 days) with medium changes every 48 h. 3 days prior to co-incubation, cultures of *G. duodenalis* NF strain trophozoites were axenically cultured in KM medium supplemented with 10% heat-inactivated FBS at 37°C to confluence. Parasites were ice-chilled for 15 min, harvested by centrifugation for 10 min at 1,300 × *g* (4°C), and resuspended in Caco-2 growth medium supplemented with bovine bile (0.6 g/L). For co-culture experiments, trophozoites were seeded at a multiplicity of infection (MOI) of 10:1. Recombinant BSHs were then added to co-cultures at different concentrations and the plates were incubated at 37°C and 5% CO_2_. After 20 h of incubation, trophozoites were collected after chilling of plates on ice and the parasite load was determined using hemocytometer (flagella mobility was used as viability criteria).

### Scanning electron microscopy

For scanning electron microscopy (SEM), fresh cultures of *G. duodenalis* trophozoites WB6 strain were treated with either rBSH47 (0.5 μg/ml), rBSH56 (0.08 μg/ml), or DCA (0.1 g/L and 0.2 g/L) in KM supplemented with 10% heat-inactivated FCS (10%), with or without bovine bile (0.6 g/L) supplementation. Cultures of treated and untreated *Giardia* trophozoites were subsequently seeded in 12 wells plates on poly-lysine glass coverslips placed at the bottom, and parasites were let to settle on the glass coverslips. After 16 h incubation, the supernatants were removed gently and cells were fixed on the glass coverslips with cacodylate 0.1 M and glutaraldehyde 2.5% (pH 7.2) overnight at 4°C. After two washing steps with 0.1 M cacodylate (pH 7.2), cells were dehydrated in a graded ethanol series (50, 70, 90, and 100%) and critical point-dried in liquid CO_2_ (Emitech K850, Quorum Technologies). Coverslips were then mounted onto holders and coated with 20 nm of gold (JEOL Fine Coater JFC-1200). The samples were then examined with a Hitachi Scanning Electron SU3500 Premium.

### Experimental infection model

OF1 mice were obtained from Charles River (Saint-Germain-Nuelles, France). Mice were housed in pathogen-free conditions and all experiments were performed under a laminar flow hood. Neonatal (suckling) mice were challenged with 10^5^ *G. duodenalis* WB6 trophozoites at day 10 by intragastric gavage (100 μl). Recombinant BSH47 was diluted in DMEM with NaHCO_3_ 16.4% (vehicle) and daily administered by intragastric gavage to neonatal mice from days 10 to 15. Control animals received vehicle instead of rBSH47. Animals were sacrificed by cervical dislocation at day 16 (peak of infection, as determined in parallel assays) and assayed for the presence of *G. duodenalis* trophozoites in the small intestine. Small intestines were resuspended in 5 ml of cold PBS, incubated on ice for 10 min, and mixed thoroughly. The parasite load was estimated using hemocytometer chambers. Mice with no detectable trophozoites (threshold: <10^3^ parasites/5 ml intestine suspension) were considered as parasite-free. All protocols were carried out in accordance with the institutional ethical guidelines of the ethics committee ANSES's Animal Health Laboratory at Maisons-Alfort on the campus of the French National Veterinary School of Alfort (ENVA), which approved this study.

### Statistical analysis

Data analysis was performed with Prism 5 software (GraphPad). One-way ANOVA, Mann-Whitney, and *t*-test were used to evaluate difference between means. Results were presented as means ± standard error of the mean (SEM). Statistical significance was calculated at a P value of 0.05 and 95% confidence interval.

## Results

### *In silico* analysis of *L. johnsonii* La1-BSHs protein sequences

The amino acid sequences of *L. johnsonii*-BSH12, BSH47, and BSH56 enzymes were blasted against reported sequences from several Gram-positive bacteria using Blastp. For the three *L. johnsonii* La1 BSH, results indicated high identity levels with BSH of different *Lactobacillus* species ranging from 54 to 100% but lower levels of identity (less than 54%) with BSH from *Bifidobacterium* and *Clostridium* species. In particular, the *L. johnsonii*-BSH12 shared 54, 57, 60, 79–84, and 60–100% identities with BSHs from *C. perfringens, L. acidophilus, L. reuteri, L. gasseri*, and *L. johnsonii*, respectively. *L. johnsonii-*BSH47 shared 54–55, 56–58, 60–66, 57, 70, and 97–100% identities with BSHs from *L. crispatus, L. reuteri, L. gasseri, L. acidophilus, L. amylovorus*, and *L. johnsonii*, respectively. Finally, *L. johnsonii*-BSH56 showed 94 and 99% identity with BSHs from *L. gasseri* and both *L. acidophilus* and *L. johnsonii*, respectively.

**Figure 1:**
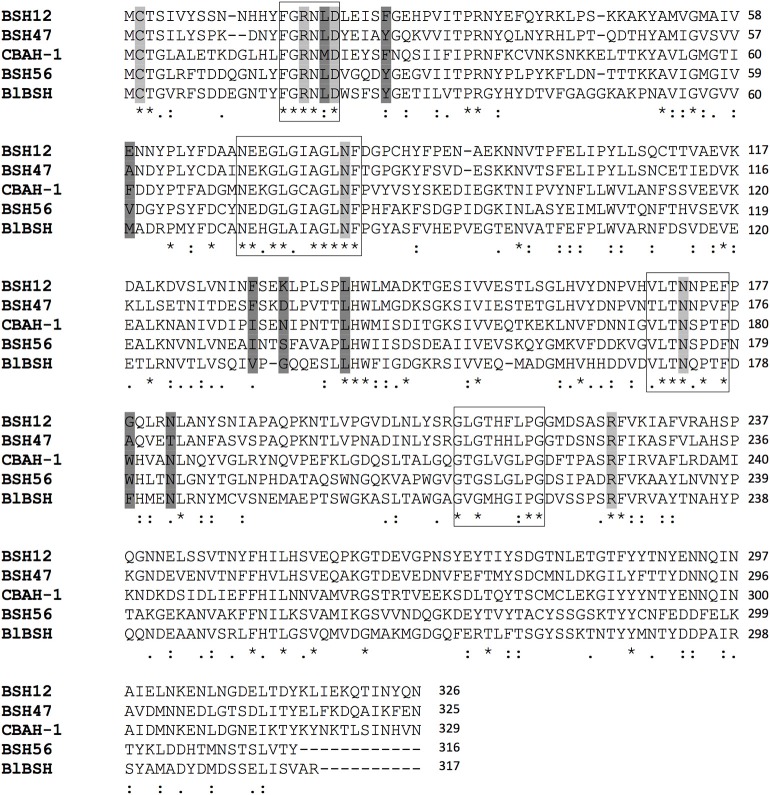
*L. johnsonii* La1 BSH possess conserved key amino acids in their predicted active sites. Multiple sequence alignments of BSH were performed with the ClustalO program (http://www.ebi.ac.uk/Tools/msa/clustalo/) using GONNET 250 matrix. An “^*^” (asterisk) indicates positions of fully conserved residues in all five sequences; a “:” (colon) indicates conservation of amino acid with strongly similar chemical properties (Gonnet PAM 250 matrix score > 0.5); a “.” indicates conservation of amino acid with similar chemical properties (Gonnet PAM 250 matrix score ≤ 0.5). Residues highlighted in light gray correspond to the predicted key active site amino acids, based on the 3D structures of BSHs from both *C. perfringens* (CBAH-1, PDB: 2BJF) (Rossocha et al., 2005) and *B. longum* (BlBSH, PDB: 2RF8) (Kumar et al., 2006). Residues highlighted in dark gray indicate amino acids putatively involved in substrate binding based on CBAH-1 3D structure (Rossocha et al., 2005; Ridlon et al., 2006). Boxes indicate conserved signatures, i.e., ^16^FGRNXD, ^72^NEXGLXXAGLNF, ^170^VXXLTNXPXF, and ^213^GXGXGXXGXPGD (CBAH-1 numbering).

The 3D structures of CBAH-1 from *C. perfringens* (Rossocha et al., 2005) and BlBSH from *B. longum* (Kumar et al., 2006) have been determined (PDB: 2BJF and PDB: 2RF8, respectively), revealing the presence of key residues in the enzymatic active site (Cys-2, Arg-18, Asp-21, Asn-82, Asn-172, and Arg-225; numbering referring to CBAH-1). In addition, experimental studies validated the importance of Arg-18 in the catalytic site (Fang et al., 2009; Lin, 2014; Lin et al., 2014). Therefore, multiple amino acid sequence alignments of BSH12, BSH47, and BSH56 with CBAH-1 and BlBSH were performed using ClustalO program (GONNET PAM 250 matrix), thereby indicating that these key residues were indeed highly conserved in all three *L. johnsonii* La1-BSHs (Figure 1). Moreover, motifs surrounding these key amino acid positions were also found to be well conserved such as ^16^FGRNXD, ^72^NEXGLXXAGLNF, ^170^VXXLTNXPXF, and ^213^GXGXGXXGXPGD, a point that has been also reported in other studies (Elkins et al., 2001; Kim and Lee, 2008). However, residues, which are predicted to be involved in the substrate-binding site based on the 3D structure of *C. perfringens*, did not appear to be conserved in either *L. johnsonii* BSH enzyme (Ridlon et al., 2006).

Predicted tridimensional structures of *L. johnsonii* BSH12, BSH47, and BSH56 were modeled with I-TASSER software, using existing 3D structures (Figure S1, BSH47 and BSH56 and Figure S2, BSH12). *Clostridium perfringens* CBAH-1 was used as a template for BSH47 modeling (RMSD: 0.64; TM-score: 0.991; Identity, 35.4%) and BSH12 (RMSD: 1.5; TM-score: 0.967; Identity: 37.3%). *Bifidobacterium longum* BlBSH was used as a template for BSH56 modeling (RMSD: 1.16; TM-score: 0.966; Identity: 43.8%). A superimposition of BSH47 and BSH56 models revealed very similar structures. In particular, the distinctive α*ββα*-folding pattern is conserved in both proteins (Patel et al., 2010; Lin et al., 2014). In addition, the residues involved in the catalytic site are superimposed which confirms a conservation of 3D structure despite a high variability of amino acid sequence among BSHs. Similar results were obtained with BSH12 (Figure S2).

### Heterologous expression and purification of bshs in *E. coli*

**Figure 2:**
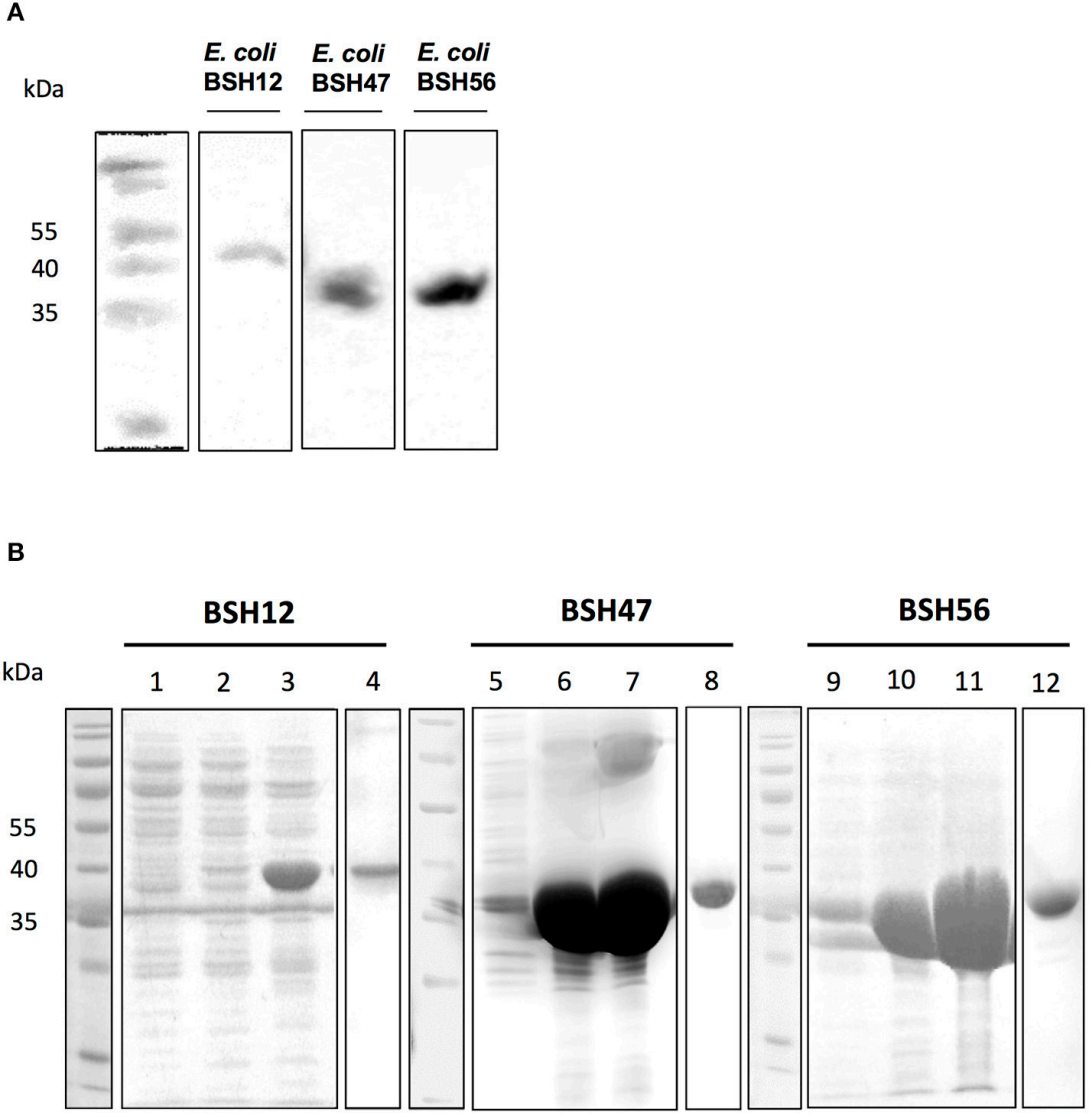
Expression and purification of *L. johnsonii* La1 BSHs. **(A)** Detection of heterologous production of BSH in *E. coli* by Western Blotting using anti-His tag antibody. Production of BSH was done under the control of pStaby system in *E. coli* (induced with 1 mM IPTG). Cytoplasmic fractions were extracted from *E. coli* SE1 harboring pLB490 plasmid (*E. coli* BSH12), pLB491 plasmid (*E. coli* BSH47) or pLB492 plasmid (*E. coli* BSH56). **(B)** SDS–PAGE analysis of purified BSHs. Purification steps of rBSH12, rBSH47, and rBSH56 with Ni-NTA affinity chromatography. Lanes 1–3 correspond to eluted fractions of rBSH12 with imidazole gradient (25, 75, and 500 mM). Lane 4 is rBSH12 after desalting. Lanes 5–7 correspond to eluted fractions of rBSH47 (imidazole gradient: 25, 75, and 500 mM); lane 8 is rBSH47 after desalting step. Lanes 9–11 correspond to eluted fractions of rBSH56 (imidazole gradient: 25, 75, and 500 mM); lane 12 is rBSH56 after desalting step.

To study the biochemical and enzymatic characteristics of *L. johnsonii* La1-BSHs, *bsh* genes (i.e., *bsh12, bsh47*, and *bsh56*) were cloned in *E. coli* CYS21 strain and expressed in *E. coli* SE1 strain. Western blot analysis from *E. coli* SE1 cells expressing His-tagged BSHs showed an efficient production of BSH12, BSH47, and BSH56, respectively (Figure 2A). High yields of heterologous proteins were produced from 1.5 L of recombinant *E. coli* cultures upon 1 mM IPTG induction. No cytotoxic effects were observed during bacterial growth. C-terminal His-tagged rBSHs were subsequently purified using Ni-NTA agarose affinity chromatography and desalted. The purity of BSHs was assessed by Coomassie-blue staining of SDS-PAGE (Figure 2B). The molecular weights observed on SDS-PAGE corresponded with those expected (based on theoretical predictions) for rBSH 47 (37.1 kDa) and rBSH56 (35.8 kDa). However, the molecular weight for rBSH12 appeared slightly higher than theoretically expected (37.4 kDa). Nanodrop quantifications of desalted proteins showed that 35 mg of rBSH47 and 28 mg of rBSH56 were successfully purified from 1.5 L of culture. However, only 3 mg of rBSH12 could be recovered.

### *L. johnsonii* La1 BSH activities and substrate specificities

**Figure 3:**
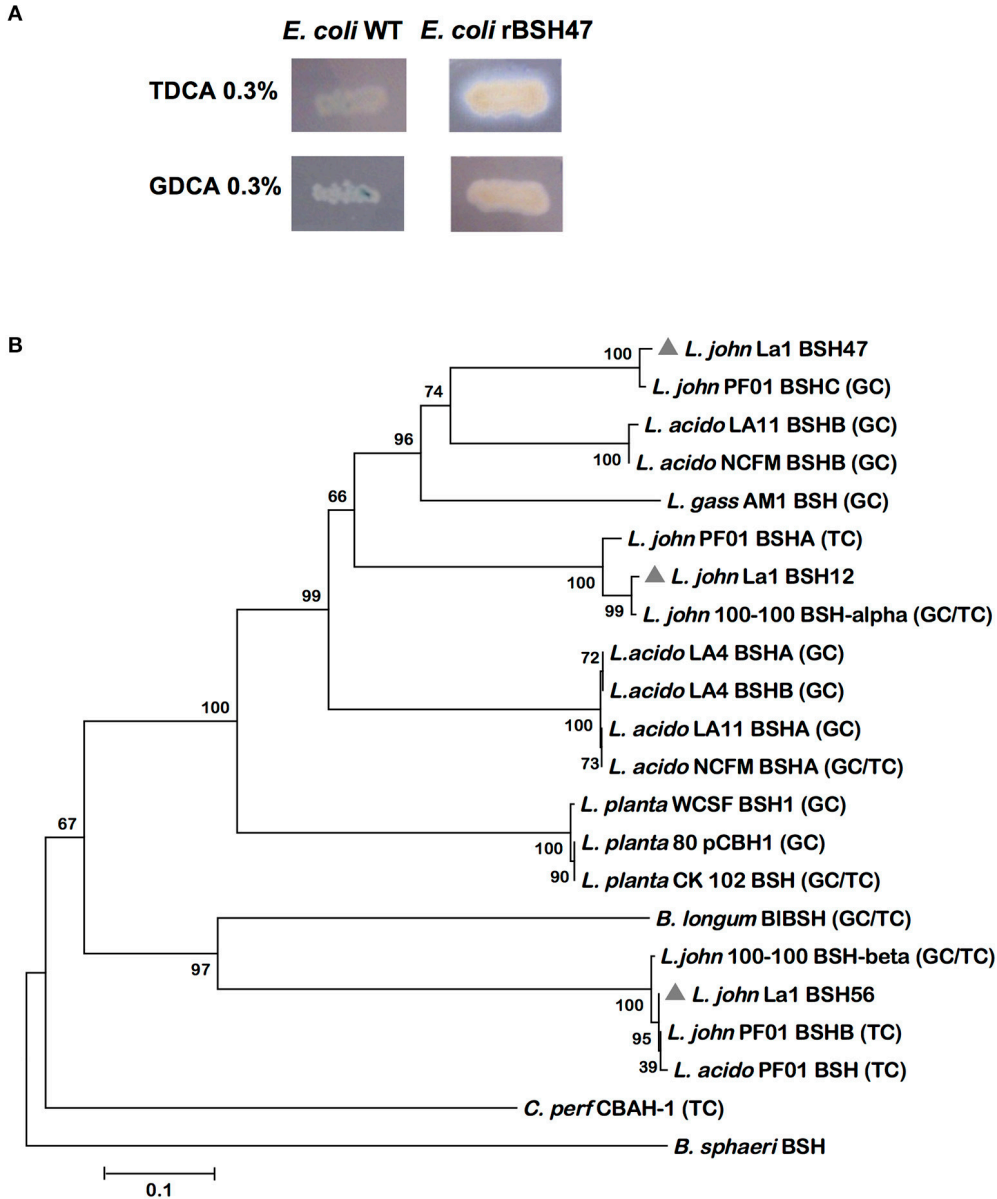
Substrate specificities of *L. johnsonii* La1 BSHs. **(A)** Hydrolase activity of rBSH47 produced in *E. coli* tested on LB plates. *E. coli* SE1 wild type or *E. coli* SE1 expressing rBSH47 were plated in the presence of taurodeoxycholic acid 0.3% (TDCA) or glycodeoxycholic acid 0.3% (GDCA) and incubated for 48–72 h. Activity is detected when a whitish halo forms around colonies. Data with *E. coli* SE1 expressing rBSH12 and rBSH56, respectively, are not shown. **(B)** Phylogenetic relationship among selected BSH sequences and substrate prediction. Programme MEGA5 was used for the phylogenetic tree and for the 500-replication bootstrap analysis. The following predicted amino acid sequences were obtained from UniProtKB/Swiss-Prot databases: BSH12, *L. johnsonii* La1 (Q74IV4_LACJO); BSH47, *L. johnsonii* La1 (Q74JG0_LACJO); BSH12, *L. johnsonii* La1 (Q74LX7_LACJO); CBAH-1, *C. perfringens* strain 13 (P54965.3); BSHA, *L. acidophilus* LA4 (ACL98173.1); BSHB, *L. acidophilus* LA4 (ACL98173.1); BSHA, *L. acidophilus* LA11 (ACL98175.1); BSHB, *L. acidophilus* LA11 (ACL98176.1); BSHA, *L. acidophilus* NCFM (YP_193782.1); BSHB, *L. acidophilus* NCFM (AAV42923.1); BSH*, L. gasseri* AM1 (ACL98172.1); BSH-α, *L. johnsonii* 100-100 (AAG22541.1); BSH-β, *L. johnsonii* 100-100 (AAC34381.1); BSHA, *L. johnsonii* pf01 (EGP12224.1); BSHB, *L. johnsonii* pf01 (EGP13287.1); BSHC, *L. johnsonii* pf01 (EGP12391.1); pCBH1, *L. plantarum* 80 (AAB24746.1); BSH1, *L. plantarum* WCSF (CCC80500.1); BlBSH, *B. longum* subsp. *longum* (2HF0); BSH, *L. acidophilus* PF01 (ABQ01980.1); BSH, *L. plantarum* CK 102 (Ha et al., 2006); Penicillin V Acylase (PVA), *Bacillus sphaericus* (3PVA). Substrate specificity, when known (see text for referenced literature) is indicated for each BSH enzyme (in brackets): (TC), specificity for tauro-conjugated bile salts; (GC), specificity for glyco-conjugated bile salts; (TC/GC), specificity for both tauro and glyco-conjugated bile salts.

The substrate specificities of the *L. johnsonii* BSHs were assayed by two approaches. Enzymatic activities were monitored in solution, using recombinant enzymes, by measuring amino acids released from the hydrolysis of conjugated bile salts as described in materials and methods. These enzymatic activity assays revealed a slight activity toward glycocholic acid, but a much higher level of activity toward taurocholic acid, for both rBSH47 and rBSH56 (Table 2). No significant enzymatic activity was detected with the purified rBSH12 with any substrate (Table 2). In parallel, the substrate specificities of the three *L. johnsonii* BSHs (produced in *E. coli*) were determined using LB agar supplemented with either taurodeoxycholic (0.3%) or glycodeoxycholic (0.3%) acids. A white and iridescent precipitate around colonies is indicative of BSH hydrolytic activity. *E. coli* SE1 strain expressing rBSH47 efficiently hydrolyzed tauro-conjugated bile salts, whereas no BSH activity was detected for glyco-conjugated bile salts (Figure 3A). *E. coli* SE1 strain expressing rBSH56 efficiently hydrolyzed both tauro- and glycol-conjugated bile salts (data not shown). A slight deconjugation was observed with *E. coli* strain producing rBSH12 against glyco-conjugated bile salts. However, BSH12 was not further tested in this study.

**Table 2:** Activities of *L. johnsonii* BSH against tauro- and glyco-conjugated bile salts.

**Enzyme**	**TDCA hydrolase activity^a^**	**Relative activity**^b^		**GDCA hydrolase activity^c^**	**Relative activity**^d^	
		U/g^*^	**%**		U/g^*^	**%**
BSH12	−	−	Nd	+	−	Nd
BSH47	++	720	100	+	65	9
BSH56	++	2600	100	++	530	21
BSH C. perf	Nd	150	26	Nd	580	100

* *μmol/5 min per g of protein*.

a *Based on activity on taurodeoxycholic acid (plate assay)*.

b *Based on activity on taurocholic acid (enzymatic assay)*.

c *Based on activity on glycodeoxycholic acid (plate assay)*.

d *Based on activity on glycocholic acid (enzymatic assay)*.

*Nd, Non-detected. One unit of BSH activity was defined as the amount of enzyme that can liberate 1 μmol of amino acid from a given substrate in 5 minutes*.

Phylogenetic relationship among selected BSH sequences and related substrate predictions were represented on a neighbor-joining tree, constructed using amino acid sequences of BSHs whose substrate specificities have been previously characterized, with 500 bootstrap replications using MEGA5 software (http://www.megasoftware.net/). Such a phylogenetic analysis showed that *L. johnsonii* La1-BSH12 is more closely related to *L. johnsonii* 100-100-BSH-α (Figure 3B), an enzyme which is able to hydrolyze both tauro- and glyco-conjugated bile salts. The *L. johnsonii* La1-BSH56 is phylogenetically related to a compact cluster including *L. johnsonii* PF01-BSHB (LjBSHB), *L. acidophilus* PF01-BSH (LaBSH), and *L. johnsonii* 100-100-BSH-β (LjBSH-β). Both BSH56 and LjBSH-β display broad substrate specificity, with a slight preference for tauro-conjugated over glyco-conjugated bile salts, whereas LaBSH and LjBSHB exclusively hydrolyze tauro-conjugated bile salts (Chae et al., 2013). Finally, *L. johnsonii* La1-BSH47 was more closely related to *L. johnsonii* PF01-BSHC (LjBSHC) hydrolyzing only glyco-conjugated bile acids, whereas BSH47 displays a preference for tauro-conjugated substrates. These observations suggest that substrate specificities are not systematically conserved among lactobacilli BSHs, despite a good conservation of their 3D-structures and of key amino acids in their active sites, which makes substrate specificity prediction based on phylogenetic analysis not straightforward for the moment. Besides, enzymatically active BSHs from *L. johnsonii* La1 that could be measured here showed a higher activity for tauro-conjugated than glyco-conjugated substrates, whereas a clear preference has been observed for glyco-conjugated bile salts among other lactobacilli-BSHs characterized so far (Tanaka et al., 1999).

### Enzymatic activities of *L. johnsonii* La1 BSH47 and BSH56 display anti-giardial effects

**Figure 4:**
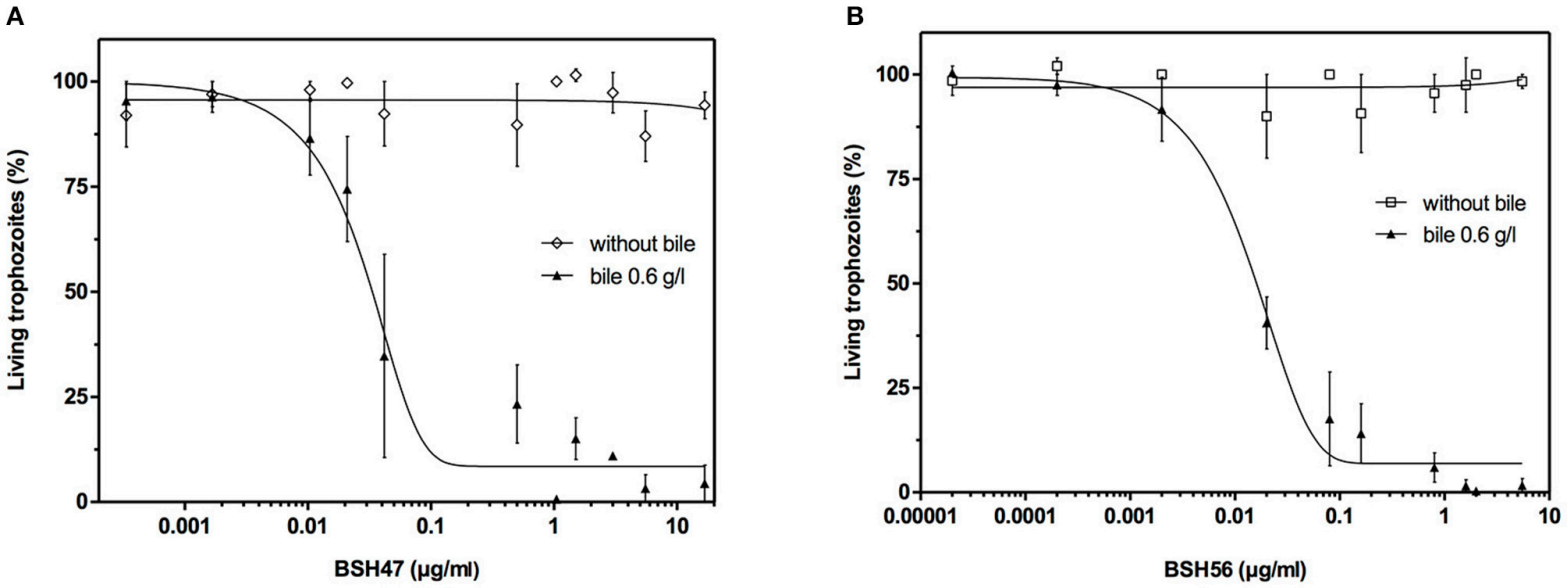
Anti-giardial activity of *L. johnsonii* La1 BSHs *in vitro*. Inhibition of *G. duodenalis* WB6 strain growth by enzymatically active rBSH47 **(A)** and rBSH56 **(B)** from *L. johnsonii* La1 with and without bovine bile supplementation (0.6 g/L) after 22 h of incubation. Increasing doses of BSH were tested ranging from 2 × 10^−5^ to 17.4 μg/ml. Values are in percentage ± SEM (standard error of the mean). Growth inhibition curves of *G. duodenalis* were calculated using Prism 5 software (GraphPad). IC50_BSH56_ = 0.018 ± 0.002 μg/ml; IC50_BSH47_ = 0.030 ± 0.003 μg/ml.

To evaluate the anti-giardial potential of purified *L. johnsonii* La1-BSHs, *G. duodenalis* WB6 trophozoites were incubated for 22 h in the presence of increasing concentrations of rBSH47 and rBSH56 from 2 × 10^−5^ to 17.4 μg/ml (rBSH12 was not tested for anti-giardial activity due to weak BSH-activity). Positive controls with *C. perfringens* BSH (Sigma-Aldrich) and negative experimental controls were set up in each independent inhibition assay. Treatments of *Giardia* trophozoites with BSHs showed a dose-dependent inhibition of the parasite growth in presence of bile, when compared to the controls without bile (Figures 4A,B). The IC_50_ of rBSH56 (IC_50BSH56_ = 0.018 ± 0.002 μg/ml) was slightly lower than that of rBSH47 (IC_50BSH47_ = 0.030 ± 0.003 μg/ml). Concentrations higher than 1 μg/ml of either rBSH47 or rBSH56, respectively, were sufficient to kill 100% of trophozoites in 22 h. Co-cultures of trophozoites and rBSHs in a growth medium without bile supplementation did not exhibit any toxic effects, further supporting the fact that the anti-giardial effect is mediated by BSH activity and requires the presence of an appropriate substrate (bile).

**Figure 5:**
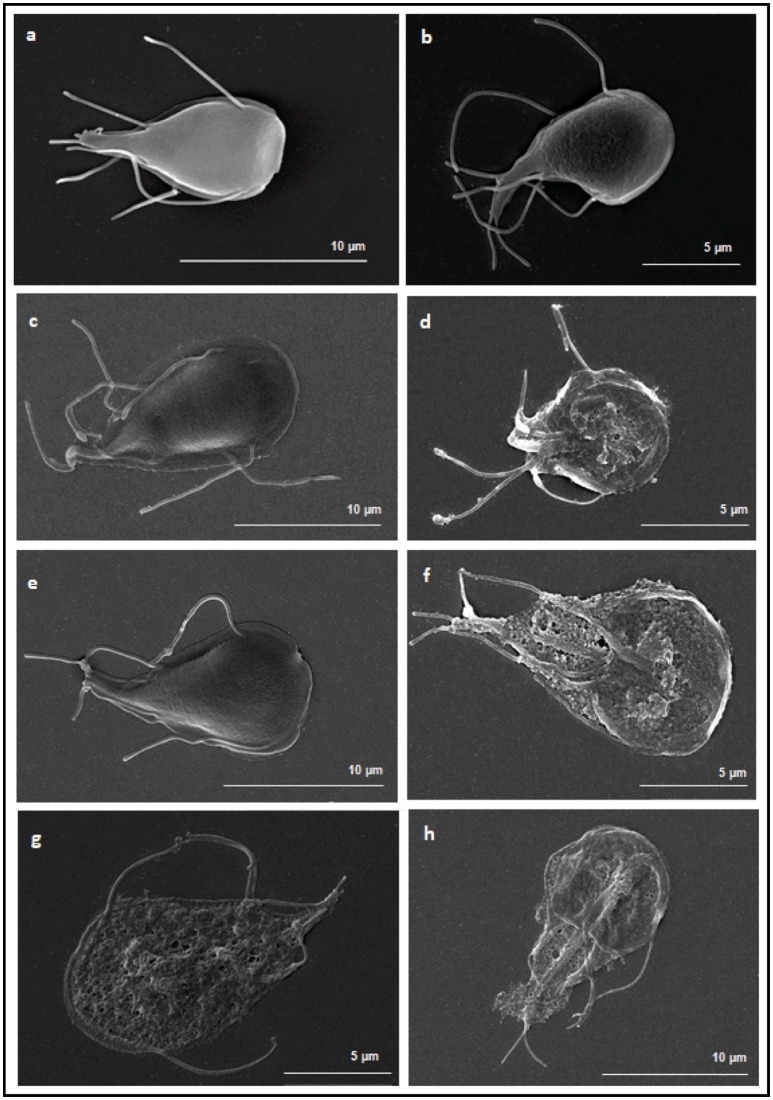
Morphological alterations following *in vitro* treatments of *G. duodenalis* by BSH or deoxycholic acid (DCA). Scanning electron microscopy of *G. duodenalis* trophozoites WB6 strain treated with either BSH47 (0.5 μg/ml), BSH56 (0.08 μg/ml) or DCA (0.1 and 0.2 g/L). **(a)** KM control (KM+ 10% FCS) and **(b)** KM control with bile (bovine bile 0.6 g/l) show the characteristic pear-shaped of trophozoites. **(c)** *G. duodenalis* treated with rBSH47and **(d)** *G. duodenalis* treated with rBSH47 with bile reveal altered morphology and plasma membrane disruption in presence of bile. **(e)** *G. duodenalis* treated with rBSH56 and **(f)** *G. duodenalis* treated with rBSH56 with bile showed similar cell lysis. **(g,h)** DCA-treated (0.1 and 0.2 g/l, respectively) *Giardia* present similar alterations and a disruption of plasma membrane exposing cell interior. Scale bar = 5 μm **(b,d,f,g)** or 10 μm **(a,c,e,h)**.

To better characterize the damages induced by BSHs activity, the morphology of *G. duodenalis* trophozoites WB6 was analyzed by SEM after 16 h of treatment with either rBSH56 (0.08 μg/ml), rBSH47 (0.5 μg/ml), or deoxycholic acid (DCA, 0.1 and 0.2 g/L), which is a major product of bile hydrolysis. SEM analysis of non-treated trophozoites showed the characteristic giardial tear-drop shape with no apparent sign of morphological alteration (Figures 5a,b). Trophozoites treated with either DCA, or rBSH56 or rBSH47 in presence of bile revealed significant structural damage when compared to controls (Figures 5c–h). BSH-treated parasites displayed several alterations such as protrusions and perforations at the surface of their plasma membrane (Figures 5d,f–h). In DCA-treated trophozoites, membrane and median body were dramatically disrupted (Figures 5g,h). In contrast, with both treatments, the ventral disk microtubule array was still observable.

### Assessment of *in vivo* anti-giardial activities of *L. johnsonii* La1 BSH47

**Figure 6:**
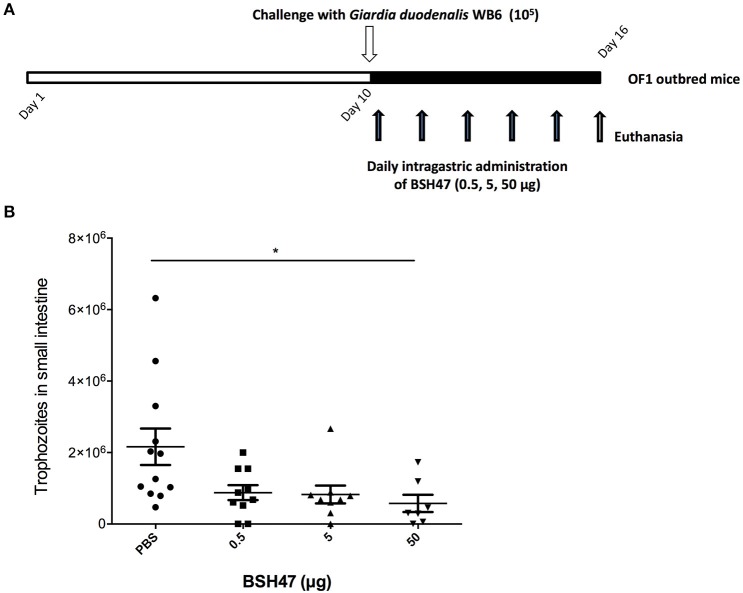
Anti-giardial activity of rBSH47 *in vivo*. **(A)** Experimental design of *G. duodenalis* infection in OF1 suckling mice model. Increasing doses of rBSH47 were daily administered by intragastric gavage (0.5, 5, 50 μg) to OF1 suckling mice from day 10 to 15. Control animals received PBS+ NaHCO_3_ 16.4% (vehicle). Mice were challenged with *G. duodenalis* WB6 trophozoites (10^5^) at day 10 by intragastric gavage. Animals were euthanized by cervical dislocation at day 16. **(B)** *G. duodenalis* trophozoites enumeration in small intestine after rBSH47-treatement. Group I (control): mice received vehicle (*n* = 12); Group II: mice received 0.5 μg of BSH47 daily for 5 days (*n* = 10); Group III: mice received 5 μg of BSH47 daily for 5 days (*n* = 9); mice received 50 μg of BSH47 daily for 5 days (*n* = 7). Small intestines were resuspended in PBS and trophozoites were counted using a hemocytometer. Values are mean ± SEM; *p* < 0.0001.

Numerous studies have reported that bile acids conjugated to taurine are predominant in mice (Claus et al., 2011). Recombinant BSH47 efficiently hydrolyzed tauro-conjugated bile acids, and its efficacy against *Giardia* has been demonstrated *in vitro*. Since this enzyme was available in larger quantities compared to rBSH56 and rBSH12, rBSH47 was selected to evaluate the potential of rBSH to treat giardiasis in a murine model (Figure 6A). OF1 suckling mice were divided into four groups (*n* = 7–12). Mice were challenged with *G. duodenalis* WB6 trophozoites (1 × 10^5^) at day 10 by intragastric gavage. Increasing doses of rBSH47 corresponding to 0.5, 5, and 50 μg (50 μl, diluted in NaHCO_3_ 16.4%) were thawed and daily administered by intragastric gavage to neonatal mice from day 10 to 15. The control group received vehicle (PBS + NaHCO_3_ 16.4%). Animals were sacrificed at day 16, corresponding to the peak of trophozoite colonization, and small intestinal contents were sampled and analyzed. Six days after inoculation, trophozoites were able to efficiently colonize and persist in the small intestine with a parasite load 20-fold higher than the inoculum (Figure 6B). In groups treated with rBSH47, the parasite burden decreased in a dose-dependent manner (Figure 6B). Interestingly, the highest dose of rBSH47 (50 μg daily for 5 days) induced a significant reduction of 68.8% of *G. duodenalis* trophozoites compared to the control group.

## Discussion

*L. johnsonii* La1 is a probiotic strain with pathogen inhibition and host immunomodulation properties (Vidal et al., 2002; Cruchet et al., 2003; Pridmore et al., 2008). The activity of BSH and lactobacilli's bile resistance have been widely accepted as key factors for gut persistence and colonization by these bacteria (Tannock et al., 1994; Tanaka et al., 2000; Begley et al., 2006). Three *bsh* and two bile acid transporters genes were identified in the genome of *L. johnsonii* La1 (Pridmore et al., 2004). In this study, we cloned, purified, and characterized these 3 BSH enzymes in order to assess their antiprotozoal effect on *Giardia*. Nucleotide homology comparisons previously highlighted the similarities between *L. johnsonii* La1 *bsh*12 and *bsh*56 with *cbsH*α and *cbsH*β from *L. johnsonii* 100-100, respectively (Elkins and Savage, 1998; Elkins et al., 2001; Pridmore et al., 2004). A neighbor-joining tree of various BSHs protein sequences from several lactic acid bacteria confirmed that *L. johnsonii* La1-BSH enzymes are phylogenetically related to BSHs from other species. In addition, they share a high degree of similarity to various sub-groups of BSHs; for instance, BSH12 shares 99% identity with *L. johnsonii* 100-100 cbsHα and *L. johnsonii* PF01 BSHA at amino acid level, and BSH56 shares 99% identity with *L. johnsonii* 100-100 cbsHβ and 98% with *L. johnsonii* PF01 BSHB at amino acid level. *L. johnsonii* La1 and *L. johnsonii* PF01 both possess a third BSH gene, *bsh*47 and *bsh*C, respectively, which is absent from *L. johnsonii* 100-100. Besides, the close relationship among *L. johnsonii* La1 BSH47, *L. johnsonii* PF01 BSHC, and *L. acidophilus* NCFM BSHB, at amino acid level, suggests that these enzymes likely share a common ancestor. These observations contribute to the idea that BSH might have been acquired through horizontal gene transfer from microorganisms sharing the same intestinal environment (Corzo and Gilliland, 1999; Franz et al., 2001; McAuliffe et al., 2005; Begley et al., 2006), although this latter hypothesis remains to be tested.

Multiple amino acid sequence alignment of *L. johnsonii* La1 BSHs indicated a high variability among characterized BSHs; however, well-conserved motifs were observed around residues involved in the active site (Cys-2, Arg-18, Asp-21, Tyr-82, Asn-172, and Arg-225). These observations are in agreement with previous studies (Rossocha et al., 2005; Fang et al., 2009; Lin et al., 2014) and highlight that the biological functions of BSHs are strictly conserved despite sequence/phenotypic variabilities. BSHs (EC 3.5.1.24) belong to N-terminal nucleophilic (Ntn) hydrolases, a superfamily of enzymes containing N-terminal cysteine residue involved in the catalytic site (Suresh et al., 1999; Kim et al., 2004). Penicillin V acylases (EC 3.5.1.11), which are closely related to BSH, also belong to Ntn hydrolases and share similar structures. *In silico* modeling, BSH47, BSH56, and BSH12 showed that the typical α*ββα* tertiary structure arrangement, which is characteristic of Ntn superfamily, is conserved (Oinonen and Rouvinen, 2000; Patel et al., 2010; Lin et al., 2014). Taken together, these structural observations indicate that the folding of BSHs remained stable during evolution despite low sequence identity, which suggest a high evolutionary pressure to maintain their functionality (Chothia and Lesk, 1986; Sander and Schneider, 1991; Krieger et al., 2003).

Experimental determination of BSH activities showed that at least 2 of the 3 BSHs from *L. johnsonii* La1 have broad substrate specificities. The enzyme BSH56 exhibited high hydrolysis activities toward tauro- and glyco-conjugated bile salts, with a preference toward tauro-conjugated substrates. Interestingly, BSH56 belongs to a phylogenetic cluster of BSH enzymes exhibiting activity exclusively directed toward tauro-conjugated bile acids. Similarly, BSH47 was more efficient at hydrolyzing tauro-conjugated bile salts and exhibited a very low relative activity toward glyco-conjugated bile salts. Surprisingly, the amino acid sequence of BSH47 is more closely related to that of *L. johnsonii* BSHC that has been reported to hydrolyze glyco-conjugated bile salts (Chae et al., 2013). Our results, therefore, contrast with previous studies reporting that most BSH enzymes isolated from lactobacilli are very efficient at hydrolyzing glyco-conjugated bile salts (Coleman and Hudson, 1995; Liong and Shah, 2005). In addition, these observations point out the limits of substrate predictions based on phylogenetic relationships considering whole enzyme sequences. The mechanisms responsible for substrate specificity of BSH enzymes are still unclear. Putative amino acids have been associated with substrate binding pockets in *C. perfringens* (Rossocha et al., 2005; Ridlon et al., 2006), but these residues are different in all 3 BSHs from *L. johnsonii*. Several studies emphasized that BSH preferably recognize conjugated substrates at amino acid moieties (Coleman and Hudson, 1995; Tanaka et al., 2000; Kim et al., 2004; Rossocha et al., 2005), whereas others still suggest that steroid moieties are recognized in priority (Moser and Savage, 2001; Begley et al., 2006; Patel et al., 2010) which could explain broad substrate specificities. Experimentally solving the 3D structures of rBSH47 and rBSH56 harboring appropriate substrates would certainly provide invaluable information regarding this important question.

The third BSH isolated from *L. johnsonii* La1, BSH12, was successfully expressed in *E. coli* and clearly observed in SDS-PAGE and by Western blotting, but no specific activity could be detected toward either substrate in liquid tests. However, a slight positive signal was observed with *E. coli* strain-expressing rBHS12 on agar plates supplemented with glyco-specific bile salts, suggesting a weak glyco-specific activity. Similar difficulties to express recombinant BSHs have been observed with BSHs from *L. plantarum* JPP2 (Ren et al., 2011) and BSH2 from *L. plantarum* WCFS1 (Lambert et al., 2008). Proteolytic degradation and misfolding of the recombinant protein produced in *E. coli* may have affected the enzyme's function (Baneyx and Mujacic, 2004). The functionality of BSH12 is, therefore, still under investigation.

As mentioned previously, the putative natural role of BSHs is to decrease the toxicity of conjugated bile salts for bacterial cells (De Smet et al., 1995). In this work, we assessed the anti-parasitic activity of recombinant BSHs and we demonstrated that rBSH47 and rBSH56 were highly active against viable trophozoites of *G. duodenalis* strains WB6 and NF. The minimum concentration found to kill 100% of *G. duodenalis* WB6 trophozoites *in vitro* was 1 μg/mL for both rBSH47 and rBSH56. When tested on human enterocyte Caco-2 cells, both rBSH47 and rBSH56 inhibited the growth of *G. duodenalis* NF in the presence of bile (0.6 g/L) in a dose-dependent fashion and prevented the attachment of the parasites to the cell monolayers (Figure S3). The rBSH56 was more effective than rBSH47 in killing both *G. duodenalis* WB6 and NF strains, although it induced more cell damages to Caco-2 cells at high concentrations (Figure S3). Recombinant BSH47 was, therefore, chosen to assess the *in vivo* effectiveness of BSH to treat *Giardia* infection in a suckling murine model. Moreover, given that bile acids are predominantly conjugated to taurine in mice (Claus et al., 2011), the ability of BSH47 to preferentially hydrolyze tauro-conjugated bile salts seemed more appropriate. We observed that rBSH47 inhibited *Giardia* growth in a dose-dependent manner *in vivo*, in keeping with the data obtained *in vitro*. At the highest dose (50 μg/mice/day) administered daily for 5 days, the parasite (trophozoite) burden was significantly reduced by 68.8% in the small intestine of the mice. Despite this important decrease of the parasite load, none of the treated mice were free of parasites at 16 days post-inoculation. The anti-giardial effects mediated by rBSH47 *in vivo* were, therefore, modest compared to their efficacy in the *in vitro* assays. This can be explained by a partial degradation of BSHs by acidic proteases and peptidases in the stomach. The activity of BSHs correlates with the amount of conjugated bile salts released in the duodenum, which is highly variable in neonates (Heubi et al., 2007). The production of deconjugated bile salts at inhibiting levels is, therefore, dependent to the bioavailability of BSH in the small intestine.

It has been reported that bile salts, and more specifically conjugated bile salts, have growth promoting effects on *G. duodenalis in vivo* (Halliday et al., 1988). Indeed, bile uptake contributes to cholesterol and exogenous phospholipids needs, which are essentials for parasite growth (Yichoy et al., 2011). So far, to our knowledge, there is no evidence showing that *Giardia* is able to deconjugate bile salts. Conjugated bile salts are, thus, directly consumed by *Giardia* without being detoxified (Farthing et al., 1985). In contrast, bacterial deconjugation aims at reducing the detergent properties of conjugated bile salts, which are more toxic for bacterial cells than secondary bile salts, i.e., cholic acid (CA), deoxycholic acid (DCA), and chenodeoxycholic acid (CDCA) (De Boever and Verstraete, 1999). It is likely that detoxification of bile salts by the duodenal microbiota has a collateral effect on parasite survival. Earlier work carried out in our lab showed that DCA and CDCA exerted cytotoxic effects on *G. duodenalis* trophozoites *in vitro* at non-micellar concentrations (Travers et al., 2016). Therefore, we investigated the morphological perturbations induced by DCA and BSHs on trophozoite cultures and we noticed both induced degenerations and perforations of the parasite plasma membrane. It has been established that DCA perturbs eukaryotic membranes structure by altering the membrane lipid microdomains (Jean-Louis et al., 2006). Furthermore, secondary bile salts induced a redistribution of cholesterol and decrease membrane fluidity. Hence, we hypothesized that secondary bile salt, and more specifically DCA, would kill *Giardia* trophozoites by damaging the cell structure. In the upper parts of the small intestine, where bile salt deconjugation occurs at high rate, the Gram-positive bacteria might be protected against secondary deconjugated bile salts by cell wall peptidoglycan.

A major side effect of BSH-based treatment would be a shift of bile salt balance in the gut. It has been reported in previous studies that an enhancement of BSH activity might impact host physiology by disturbing fat digestion and lipid metabolism (Begley et al., 2006; Lin et al., 2014). Moreover, secondary bile acids resulting from the deconjugation of bile salts have also been linked to DNA damage in bacterial and host cells, colon cancer, and inflammation (Cheah and Bernstein, 1990; Moser and Savage, 2001; Bernstein et al., 2005). On the other hand, BSH activity is a natural process that plays a central role in the reduction of cholesterol (Jones et al., 2013).

The aim of this study was to evaluate the anti-giardial potential of BSHs against *G. duodenalis*. We expressed for the first time the BSHs isolated from the probiotic strain *L. johnsonii* La1 and showed that rBSH47 and rBSH56 exhibited high specific activity and broad substrate specificities. Antiprotozoal assays demonstrated that BSHs were highly effective against *G. duodenalis in vitro* and *in vivo* and represent a promising therapeutic strategy based on their natural catalytic activity. Future studies will determine whether such treatment approaches should be of short duration in order to avoid putative side effects related to the enhancement of bile salt deconjugation. Besides, this anti-giardial effect can be extended to any BSH activity as long as it efficiently converts conjugated bile salts into their deconjugated counterparts. However, further works are still needed to investigate the positive impact of such treatment on the pathophysiology of giardiasis, including protective effects on epithelial permeability, mucosal injury, and malfunction. Moreover, newer galenic formulations are needed in order to provide a better persistence of rBSHs *in vivo*, which can improve health outcomes in routine clinical and veterinary usages.

## Author contributions

IF, PG, BP, TA, and LB-H conceived and designed the study. TA, IF, and LB-H produced and isolated the recombinant BSH, performed the biochemical characterizations with SC and the *in silico* analyses. TA, SC, AB, and IF performed the *Giardia* assays *in vitro* and SEM. TA, MT, and BP performed the *Giardia* assays in the suckling mice model. IV, PL, and PG discussed the experiments and results. TA, IF, and LB-H wrote the manuscript with contributions from all authors.

### Conflict of interest statement

The authors declare that the research was conducted in the absence of any commercial or financial relationships that could be construed as a potential conflict of interest.

## Supplementary material

Figure S1 Protein structure prediction of *L. johnsonii* La1 BSHs. Protein structures were modeled for *L. johnsonii* BSH56 **(a)** and BSH47 **(b)** using I-TASSER software (http://zhanglab.ccmb.med.umich.edu/I-TASSER/). CBAH-1 (PDB: 2BJF) from *C. perfringens* (Rossocha et al., 2005) and BlBSH from *B. longum* (Kumar et al., 2006) (PDB: 2HF0) were used as templates for modeling BSH47 and BSH56, respectively. Best values of the C-score were chosen for model structure prediction. Visualization of predicted structures for BSH47 (red, **a**) and BSH56 (blue, **b**) and superimposition **(c,d)** were performed using Pymol software (https://www.pymol.org/).

Figure S2 Protein structure prediction of *L. johnsonii* La1 BSH12. Protein structure was modeled for *L. johnsonii* BSH12 using I-TASSER software (http://zhanglab.ccmb.med.umich.edu/I-TASSER/). Bile salt hydrolase (PDB: 2RF8) from *C. perfringens* was used as a template for modeling. Best value of the C-score was chosen for model structure prediction. Visualization of predicted structure was performed using Pymol software (https://www.pymol.org/).

Figure S3 Anti-giardial effect of BSH in co-culture with Caco2 cells. To further investigate the effect of *L. johnsonii* La1-BSHs on the adherence of *Giardia* enterocytes, differentiated Caco-2 cells were co-incubated with fresh trophozoites cultures of *G. duodenalis* NF strain and treated with either rBSH47 or rBSH56, over a range of concentrations from 0.005 to 1 μg/ml. *G. duodenalis* NF strain trophozoites were inoculated to Caco2 cells at a multiplicity of infection (MOI) of 10:1 in the presence of increasing doses of rBSH47, rBSH56 or DMEM. Anti-giardial activity assays were performed with bovine bile (0.6 g/l) added to the medium. The results are expressed as relative percentage of growth compared to untreated trophozoites as negative control. Data are presented as mean ± SEM and correspond to triplicates. As expected from previous experiments done with the *G. duodenalis* WB6 strain, both BSHs triggered a growth inhibition of *G. duodenalis* NF trophozoites, in a dose-dependent fashion, after 20 h of exposure (this figure). In contrast, high concentrations of rBSH in assays induced cell damages on Caco-2 monolayer (data not shown). This phenomenon might be due to a putative cytotoxicity of deconjugated bile salt. Interestingly, neither rBSH47 nor rBSH56 displayed cytotoxic effects when tested in absence of bile (data not shown).
