# Supplementary figures and images for: Bile-Salt-Hydrolases from the Probiotic Strain Lactobacillus johnsonii La1 Mediate Anti-giardial Activity in Vitro and in Vivo
Source: Front Microbiol. 2018 Jan 31;8:2707. doi: 10.3389/fmicb.2017.02707 (PMC5810305; doi:10.3389/fmicb.2017.02707)

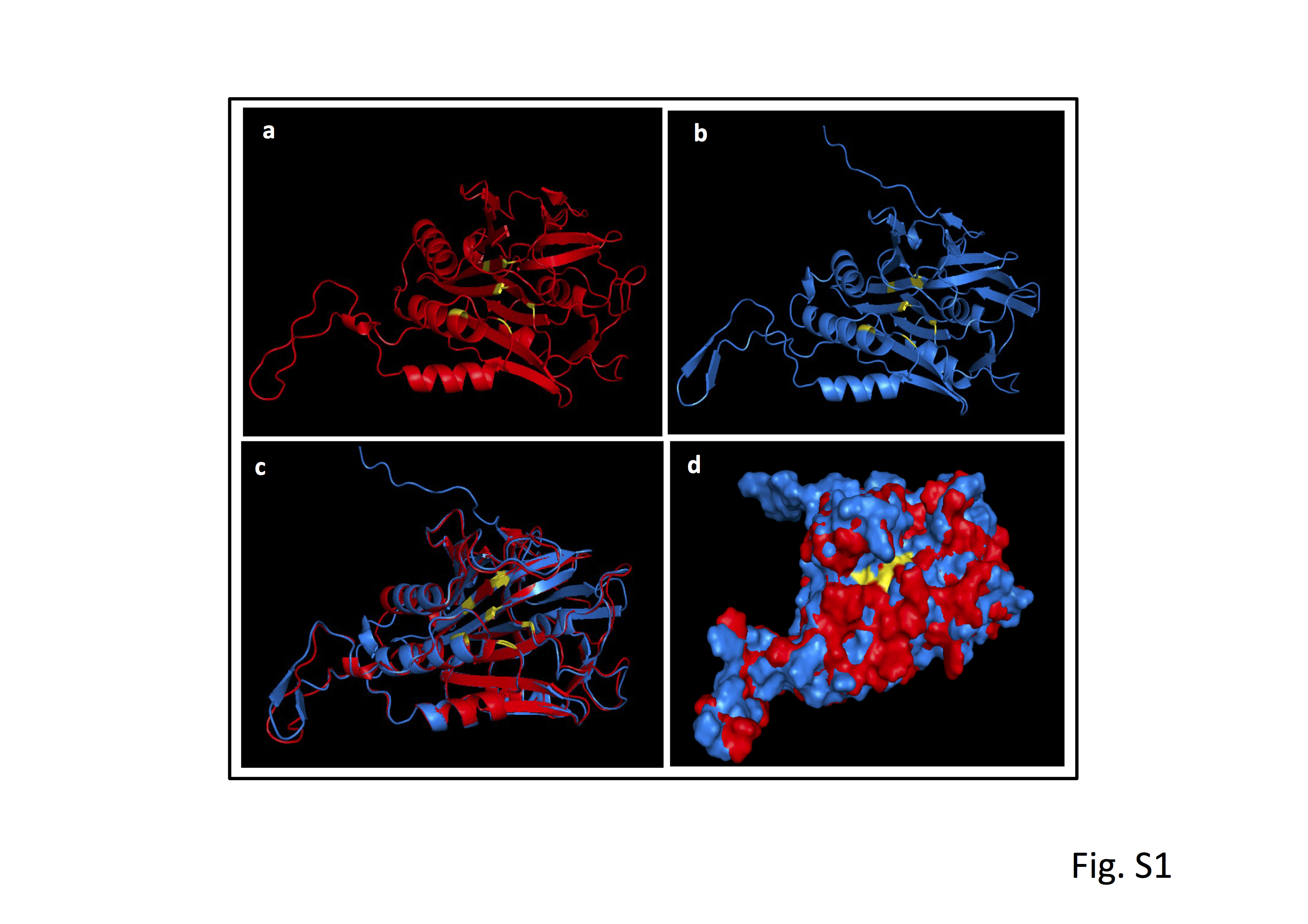

Supplement: Figure S1 — Protein structure prediction of L. johnsonii La1 BSHs. Protein structures were modeled for L. johnsonii BSH56 (a) and BSH47 (b) using I-TASSER software (http://zhanglab.ccmb.med.umich.edu/I-TASSER/). CBAH-1 (PDB: 2BJF) from C. perfringens (Rossocha et al., 2005) and BlBSH from B. longum (Kumar et al., 2006) (PDB: 2HF0) were used as templates for modeling BSH47 and BSH56, respectively. Best values of the C-score were chosen for model structure prediction. Visualization of predicted structures for BSH47 (red, a) and BSH56 (blue, b) and superimposition (c,d) were performed using Pymol software (https://www.pymol.org/). [file Image1.TIFF]

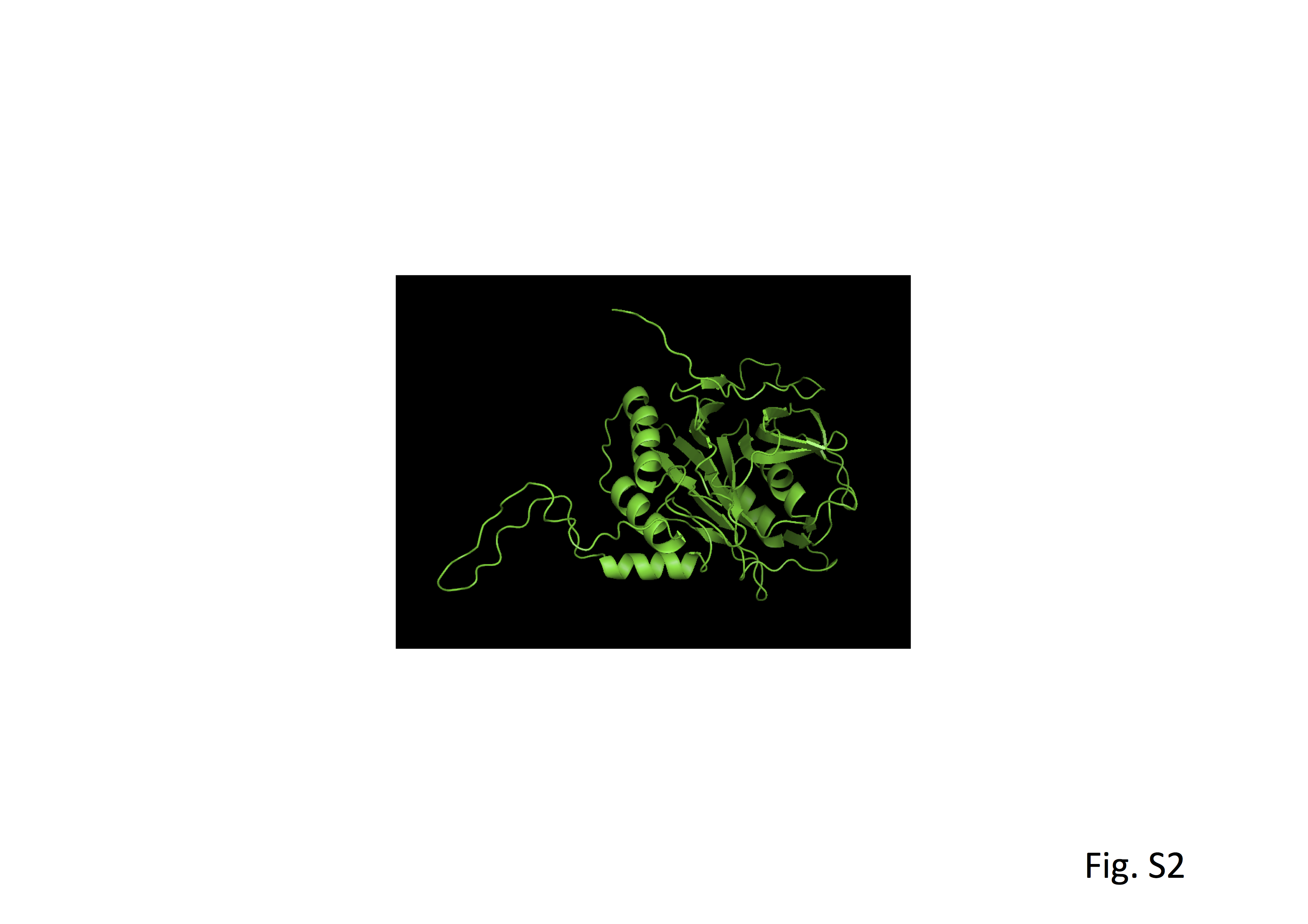

Supplement: Figure S2 — Protein structure prediction of L. johnsonii La1 BSH12. Protein structure was modeled for L. johnsonii BSH12 using I-TASSER software (http://zhanglab.ccmb.med.umich.edu/I-TASSER/). Bile salt hydrolase (PDB: 2RF8) from C. perfringens was used as a template for modeling. Best value of the C-score was chosen for model structure prediction. Visualization of predicted structure was performed using Pymol software (https://www.pymol.org/). [file Image2.TIFF]

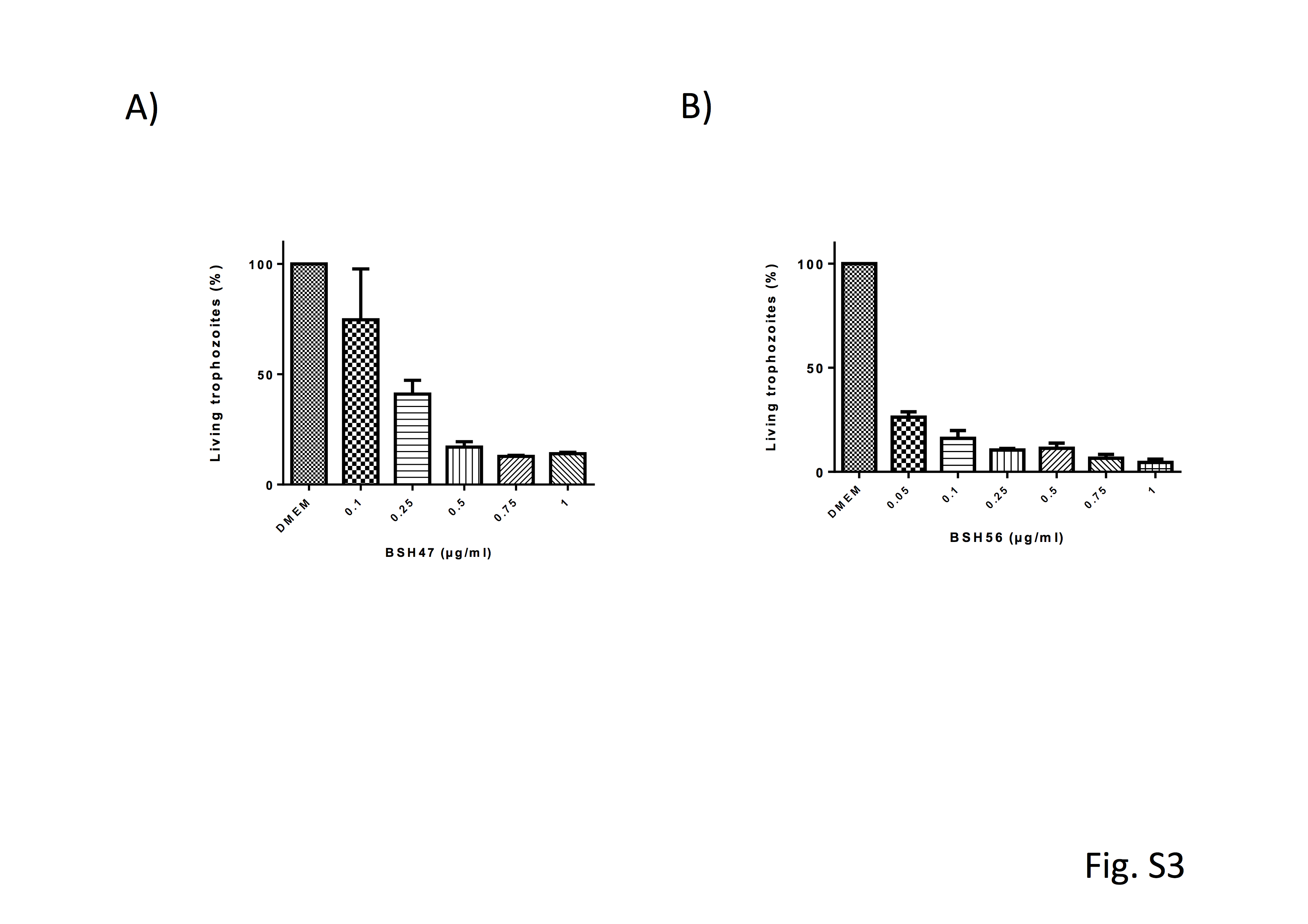

Supplement: Figure S3 — Anti-giardial effect of BSH in co-culture with Caco2 cells. To further investigate the effect of L. johnsonii La1-BSHs on the adherence of Giardia enterocytes, differentiated Caco-2 cells were co-incubated with fresh trophozoites cultures of G. duodenalis NF strain and treated with either rBSH47 or rBSH56, over a range of concentrations from 0.005 to 1 μg/ml. G. duodenalis NF strain trophozoites were inoculated to Caco2 cells at a multiplicity of infection (MOI) of 10:1 in the presence of increasing doses of rBSH47, rBSH56 or DMEM. Anti-giardial activity assays were performed with bovine bile (0.6 g/l) added to the medium. The results are expressed as relative percentage of growth compared to untreated trophozoites as negative control. Data are presented as mean ± SEM and correspond to triplicates. As expected from previous experiments done with the G. duodenalis WB6 strain, both BSHs triggered a growth inhibition of G. duodenalis NF trophozoites, in a dose-dependent fashion, after 20 h of exposure (this figure). In contrast, high concentrations of rBSH in assays induced cell damages on Caco-2 monolayer (data not shown). This phenomenon might be due to a putative cytotoxicity of deconjugated bile salt. Interestingly, neither rBSH47 nor rBSH56 displayed cytotoxic effects when tested in absence of bile (data not shown). [file Image3.TIFF]
